# Objectively measured moderate-to-vigorous physical activity does not attenuate prospective weight gain among African-origin adults spanning the epidemiological transition

**DOI:** 10.21203/rs.3.rs-5043485/v1

**Published:** 2024-12-16

**Authors:** Jessica C. Davies, Candice Choo-Kang, Larske Soepnel, Hayli Geffen, Chad Africa, Asanda Mtintsilana, Pascal Bovet, Bharathi Viswanathan, Kweku Bedu-Addo, Prince Oti Boateng, Kingsley Apusiga, Oscar Akunor Dei, Terrence E. Forrester, Marie Williams, Estelle V. Lambert, Dale E. Rae, Nandipha Sinyanya, Brian T. Layden, Jack A. Gilbert, Gertrude Ecklu-Mensah, Cara Joyce, Amy Luke, Lara R. Dugas

**Affiliations:** University of Cape Town; Loyola University Chicago; University of Cape Town; University of Cape Town; University of Cape Town; University of Cape Town; University Hospital of Lausanne; Ministry of Health; Kwame Nkrumah University of Science and Technology; Kwame Nkrumah University of Science and Technology; Kwame Nkrumah University of Science and Technology; Kwame Nkrumah University of Science and Technology; University of the West Indies; University of the West Indies; University of Cape Town; University of Cape Town; University of Cape Town; University of Illinois at Chicago; University of California, San Diego; University of California, San Diego; Loyola University Chicago; Loyola University Chicago; University of Cape Town

**Keywords:** Objectively measured physical activity, prospective weight change, obesity

## Abstract

Traditional obesity-related public health messaging often includes physical activity (PA) recommendations. However, at the population level, the data are conflicting, especially when comparing different self-reported vs measured techniques across different settings and populations. We measured the association between moderate-to-vigorous intensity PA (MVPA) and prospective weight change across five African-origin populations and the extent to which MVPA attenuated weight change over time.

At baseline, 2,500 adults (median age: 37y) were recruited into the Modelling the Epidemiologic Transition Study (METS), from Ghana, South Africa, Jamaica, Seychelles, and US. 2000 participants were followed up 8 years later, with 851 participants having complete 7-day accelerometry to measure MVPA at both time points. Generalised estimating equations were used to explore the longitudinal association between weight and MVPA adjusted for several confounders.

The obesity prevalence at baseline was 27.5% which increased to 38.0% at follow-up. Baseline MVPA varied from 7 (IQR: 4, 16) min/day in US women to 52 (IQR: 36, 78) min/day in South African men, and similarly at follow-up ranged from 8 min/day to 41 min/day among the same participant groups. While overall, engaging in higher MVPA levels was associated with a lower body weight, such that every additional 30 min of MVPA equalled a 600g lower body weight (p = 0.04), the interaction between time and MVPA was not statistically significant (p = 0.18). Therefore, regardless of the amount of MVPA at any time point, body weight increased over time.

Despite the association between MVPA and weight, our results suggest that objectively measured longitudinal MVPA was not associated with the change in 8-year weight in African-origin adults. Our research confirms that while PA is a critical determinant of cardiovascular health, it alone may not be enough to stem the rising obesity burden.

## Introduction

As of 2022, the World Health Organisation (WHO) estimates that more than 1 billion people are living with obesity worldwide [1]. While the burden has historically been highest in high-income countries, such as the United States (US), the epidemiologic transition, characterised by rapid urbanisation and nutrition transitions, has resulted in a rapidly worsening disease burden in low- and middle-income countries [2]. In fact, it is estimated that 78% of global adult deaths attributable to high body mass index (BMI) occur in low-to middle-income countries [3].

Physical activity (PA), defined as any bodily movement that results in energy expenditure [4, 5], is an important component of energy balance [6]. Consequently, the WHO has issued a global PA action plan and published new daily PA guidelines [5, 7, 8]. The guidelines distinguish between the importance of different intensities of PA, including light-, moderate-, and vigorous-intensity PA. Despite these extensive public health efforts, the global prevalence of insufficient physical inactivity has remained relatively stable [9] while obesity prevalence continues to rise. Nonetheless, specialist and government organisations advocate for increased PA to prevent weight gain [10]. To be clear, it is irrefutable that purposeful exercise or engaging in higher levels of PA is critical for improving cardiorespiratory fitness, which in turn plays an important role in reducing morbidity and mortality [5, 6, 11–14]. However, the evidence regarding the role of habitual PA in preventing obesity and promoting prospective weight loss remains conflicting [15–24]. Notwithstanding, supervised, purposeful exercise has been shown to have some weight loss benefits when either used alone [22, 23] or in combination with dietary interventions [25, 26].

Many studies exploring the relationship between weight gain and PA have relied on self-report measures, such as questionnaires, particularly at the population level, as opposed to using objective measures, such as actigraphy [16, 20, 21, 27, 28]. Compared to objectively measured PA, self-reported PA has been shown to poorly estimate total volume and/or intensity of PA owing to recall bias, and generally results in an overestimate [29, 30]. In addition, there is no consistency in the dynamics of the obesity epidemic and long-term trends in PA at a population level [17, 31]. On the other hand, studies have found that PA impacts metabolic functioning [32], and some experts argue that a decline in population-based PA levels is instrumental in the obesity epidemic [16, 18, 33].

Lifestyle changes resulting from urbanisation in low- to middle-income countries may significantly contribute to the increase in obesity worldwide [15]. Investigating the patterns of PA in populations at different stages of this transition, with varying degrees of obesity, may provide insights into the role of PA as such a risk factor [34]. The Modeling the Epidemiologic Transition Study (METS) is a well described cohort study of prospective weight change in adults of predominantly African descent from lower socio-economic communities within five countries, spanning the epidemiologic transition [10, 16]. Previously, it was shown that baseline PA is not associated with 2-year prospective weight change [16]. In the current study, the follow-up period was extended to 8 years and included change in MVPA levels. The overall aim of the current analysis is to explore the association between objective MVPA and prospective weight change in the same 5 METS cohorts.

## Methods

### Study design and settings

METS was originally initiated in 2009 to investigate the relationships between body composition, PA, prospective weight change and cardiometabolic disease risk in five African-origin populations (Ghana, South Africa, Jamaica, Seychelles, and the US). In 2017, METS-Microbiome, a follow-up study of METS, was initiated [35]. Detailed protocols from both METS and METS-Microbiome have been published [10, 35]. The Ghanaian study site is in rural Nkwantakese, home to approximately 5,000 residents. Khayelitsha, South Africa’s study site, and sixth largest township, is adjacent to the city of Cape Town and home to approximately 400,000 people [10, 36]. The Seychelles study population was recruited from Victoria which is on the main island, Mahé, and home to approximately 28,000 people. The Jamaican study site is in Spanish Town, a suburb of Kingston, the country’s capital and largest city. Maywood, the study site in the US, is a low-income African American community adjoining the western border of Chicago, Illinois [10]. These five sites were purposefully selected to represent the “epidemiologic transition” spectrum, with Ghana and the US as opposite ends of the Human Development Index (HDI) spectrum [35].

#### Recruitment and enrolment

METS enrolled approximately 2,500 adults (n = 500 per study site) between the ages of 25 and 45 years between January 2010 and September 2011. Subsequently, METS-Microbiome enrolled approximately 2000 of the original METS participants (n = 400 per study site), then aged between 35 and 55 years, between January 2017 and December 2019. Potential participants were excluded from METS, and in turn METS-Microbiome, if they had any self-reported infectious diseases (including active malaria and Human Immunodeficiency Virus (HIV)), were pregnant or lactating women, or had conditions which impair everyday physical activities, e.g., severe osteo- or rheumatoid arthritis, or any lower body disability [10, 34, 35].

The protocols for METS and METS-Microbiome were approved by the Institutional Review Board of Loyola University Chicago, IL, US; the Committee on Human Research Publication and Ethics of Kwame Nkrumah University of Science and Technology, Kumasi, Ghana; the Human Research Ethics Committee of the University of Cape Town, South Africa; the Board for Ethics and Clinical Research of the University of Lausanne, Switzerland; and the Ethics Committee of the University of the West Indies, Kingston, Jamaica. All participants provided written informed consent [10, 34, 35]. The protocol for the current analysis was also approved by the University of Cape Town’s Human Research Ethics Committee (HREC ref: 632/2022). All methods were completed in accordance with relevant guidelines and regulations.

#### Research procedures and data collection methods

As per standardised protocols, participants completed extensive questionnaires and attended the METS research sites to undergo clinical health measures and receive accelerometers for the objective measurement of PA. All data collection methods were the same at both time points (i.e., METS baseline and METS-Microbiome, hereafter referred to as “baseline” and “follow-up” respectively). All measurements were completed in the early morning [10, 37], with participants arriving at the study sites following an overnight fast.

#### Clinical measures and health history

Measurements involving weight and height were performed, after participants were asked to remove their shoes, while wearing only lightweight clothing. Weight (kg) was recorded to the nearest 0.1kg, and height (cm) was recorded to the nearest 0.1cm. Waist and hip circumferences (in centimeters) were measured to the closest 0.1cm at both the umbilicus, and the point of maximum protrusion of the buttocks, respectively. BMI was calculated from weight and height as kg/m^2^ [34]. Body composition was estimated using bioelectrical impedance analysis (BIA) using a single-frequency (50 kHz) impedance analyser (model BIA 101Q; RJL Systems, Clinton Township, MI). Fat mass (kg) was estimated using an equation previously validated in the METS cohorts which incorporated measured resistance[10, 16]. Finally, all participants completed a detailed health history questionnaire, which included socio-demographic information.

#### Physical activity measurement

The Actical accelerometer (Phillips Respironics, Bend, OR, US) was used to objectively measure PA, as has been previously described [34, 37]. Briefly, the accelerometer was worn at the level of the waist just behind the right hip, continuously over an 8-day period [34]. This period yielded six full days (i.e., 8 days of wearing the monitor with two partial days on either end of the period). The original METS study was able to determine that this amount of wear time provides a good level of reliability with an interclass correlation coefficient of 0.83–0.92 across the five sites [10]. The time period for assessing PA conducted daily was between 07:00 and 23:00, and this was done to standardise the measurements as there are no global guidelines surrounding the definition of sleep-time vs. awake-time for accelerometery data which is collected for a 24-hour period [37].

For data analysis, we first determined non-wear time defined as ≥ 90 minutes of consecutive zero activity counts by running the raw data from the accelerometers through a SAS macro programme [10]. This criterion was formed on visual inspection of the wear/non-wear patterns across a range of different string-length criteria in a subset of files from each country [37]. For a days’ entry to be valid, the measurement period should include at least 10 hours of daytime wear time, i.e., wearing the accelerometer for ≥ 62% of available wear time. A participant’s PA dataset was considered for analysis if it contained at least four valid days of PA measurement [34].

Raw accelerometer data are converted to “activity counts”, corresponding with the frequency and magnitude of acceleration, for subsequent analysis. This allows for the use of published cut-point thresholds to assess sedentary behaviour, light-, moderate- and vigorous-intensity PA from the accelerometer data as follows: sedentary behaviour < 100 counts per minute (cpm), light-intensity PA: 101–1534 cpm, moderate-intensity PA 1535–3959 cpm and vigorous-intensity PA ≥ 3960 cpm [38, 39]. The same protocol which was used for the National Center for Health Statistics analysis of accelerometery data in the National Health and Nutrition Examination Survey (NHANES) was used to define minutes [40] spent in PA intensities (and for the purposes of this study, included only sedentary, moderate, vigorous, or moderate- and vigorous-intensity PA (MVPA)), presented as the overall time in minutes combined in intervals of either 1- or 10-minutes [37]. As was used in the NHANES study protocol, the current study made allowances for up to 2 minutes of below threshold count activity before acknowledging that an activity bout had ended, and therefore the 10-minute interval should be regarded as a modified 10-minute bout [10, 37, 40]. Total activity counts divided by total wear time is used as an overall measure of daily PA intensity [37]. Average counts and time spent in 1-minute bouts of MVPA, and sedentary behaviour time are also included. For the purposes of these analyses, MVPA measured in 1-minute bouts were used.

### Statistical analysis

Data analysis was conducted using R (version 4.2.2, Posit, PBC, Boston, MA). Descriptive statistics were used to summarise participant characteristics at each of the study sites, through medians and interquartile ranges (IQR) for continuous measures and proportions for categorical variables (Table 1). All tables are sequenced by country according to their HDI ranking, from lowest (Ghana) to highest (US). PA was described as the median (IQR) total number of minutes which a person moves in a day, and then the number of minutes the person moves in each intensity category (sedentary, moderate, and vigorous). Overweight and obese were considered as categorical variables according to the established BMI cut-offs of 25–29.9kg/ m^2^ and ≥ 30kg/m^2^, respectively. Following data cleaning, including removal of participants with no valid PA data, results were stratified according to site and sex.

GEEs were used to explore the association between longitudinal weight change and MVPA over time as a main exposure. Due to its robustness in handling the within-subject correlations, GEEs are particularly suited for analysing correlated data structures typically encountered in longitudinal studies with repeated measures designs. Due to the continuous outcome variable (weight), a linear link function was used in the GEE model. An exchangeable correlation structure was used due to time being binary (i.e., baseline and follow-up). A GEE model was used with PA measured at both time points, and the model was run with and without an interaction between MVPA and time. Age, sex, site, and obesity (time varying) were included as covariates due to their relevance in testing whether PA impacts prospective weight change over the given time-period. Sensitivity analysis included fitting the same models described above but stratified by obesity status at baseline. For ease of model interpretation, MVPA per day was reported in bouts of 30 minutes/day.

## Results

### Demographic and health-related findings

Participant characteristics are presented in Table 1. At baseline, METS recruited 2,506 participants, while METS-Microbiome completed follow-up in 1,518 participants. From this, 851 participants had complete, valid PA datasets available at both time points. The descriptive characteristics for all participants, at both time-points, are provided in Supplementary Tables 1 (Baseline) and 3 (Follow-up). Of note, BMI, sex and MVPA of the final sample for the current analysis (n = 851) were all similar to those in the group without follow-up PA data (n = 1473). The median age at baseline was 37 (IQR: 31, 41) years and the median age at follow-up was 44 (IQR: 39, 49) years. Overall, the median weight increased between baseline and follow-up for both men and women and at every site except for the US site (n = 114), where weight decreased slightly for men (87 [76,109] kg vs 85 [74,101] kg) and remained stable for women (97kg [77, 111] kg and 97 [83, 113] kg).

The measures of adiposity were generally consistent with countries’ HDI ranking – the lower ranking countries had lower adiposity while the higher-ranking countries had higher adiposity. Among men, the highest median BMI was found in the Seychellois participants (28 kg/m^2^, IQR: 24, 31), followed closely by the US men (28 kg/m^2^, IQR: 24, 34). Obesity prevalence doubled from baseline to follow-up in the Ghanaian (3–6%), South African (2–4%), and Jamaican (4–7%) men. Although US participants remained the most obese population, the prevalence decreased from baseline to follow-up in this group (50–35%). Among the women, Ghanaian participants had the lowest median BMI (28 kg/m^2^; IQR: 24, 31), and the lowest obesity prevalence (35%). While women consistently had a notably higher obesity prevalence than men at each site at follow-up, more than half of the women from each country were overweight or obese. Of concern is that 74% of the US females were obese.

### Physical activity parameters

**Figure 1:**
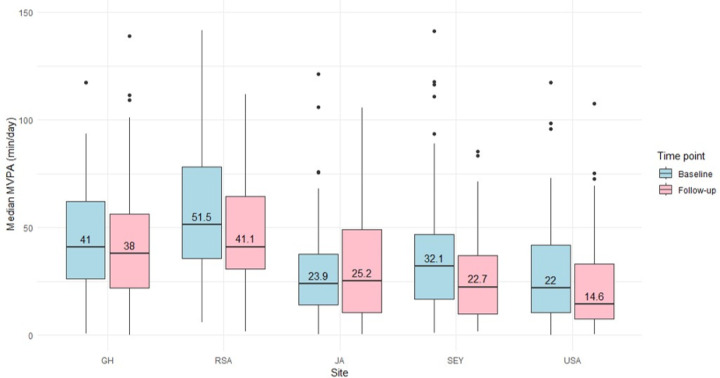
Median MVPA for men by timepoint and site (n=362) Data are presented as median, interquartile ranges, maximum and minimum. MVPA: moderate-to-vigorous intensity PA; GH: Ghana, RSA: South Africa, JA: Jamaica, SEY: Seychelles, USA: United States.

**Figure 2:**
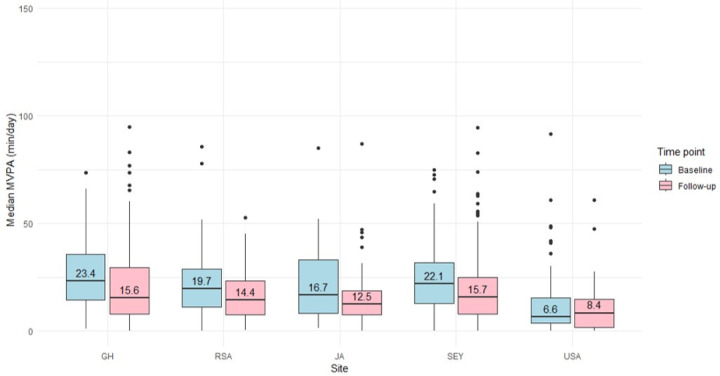
Median MVPA for women by timepoint and site (n=489) Data are presented as median, interquartile ranges, maximum and minimum. MVPA: moderate-to-vigorous intensity PA; GH: Ghana, RSA: South Africa, JA: Jamaica, SEY: Seychelles, USA: United States.

Accelerometer-derived PA data are presented in Table 2, Fig. 1 and Fig. 2. South African men recorded the highest MVPA at baseline (52 min/day; IQR: 36, 78) and follow-up (41 min/day; IQR: 31, 65), reflecting substantial time spent in this intensity of PA. in contrast, US men had the lowest MVPA at baseline (22 min/day; IQR: 11, 42) and follow-up (15 min/day; IQR: 8, 33). Among the women, Ghanaians had the highest MVPA at baseline (23 min/day; IQR: 14, 36) and follow-up (16 min/day; IQR: 8, 30), whereas women in the US had the lowest MVPA at both baseline (7 min/day; IQR: 4, 16) and follow-up (8 min/day; IQR: 2, 15).

### Associations between MVPA and prospective weight change

The GEE models evaluating the association between MVPA at both timepoints, and prospective weight change are presented in Table 3. While overall, engaging in higher levels of MVPA was associated with lower body weight (Model 1a). For every additional 30min/day spent in MVPA at a particular time point (i.e., either baseline or follow-up), weight at that timepoint was approximately 0.63kg (0.04–1.22kg decrease, p = 0.04) lower on average, when accounting for covariates such as sex, age, obesity, site and time point (Model 1b). However, the interaction term between time and MVPA was not found to be statistically significant (p = 0.16), indicating that the extent to which weight changed over time was not related to the extent to which MVPA changed over time. Additionally, on average, weight increased by 2.61kg (2.02–3.20kg increase; p < 0.001) at follow-up, irrespective of MVPA at any time point. Findings were similar when stratified by obesity status at baseline (Supplementary Tables 6 and 7).

### Associations between weight and covariate contributors to the models

Further, being male, obese, from the US, and time were all associated with weight change, adjusting for covariates (Table 3, Model 1a). On average, in participants categorised as being obese, weight was 18.17kg (16.57–19.76kg) more relative to those with non-obese BMIs (p < 0.001). Weight was 3.66kg (1.78–5.55kg) less on average in females than males (p < 0.001). On average, participants from all sites outside of the US weighed less at any time point in the study (p < 0.001) compared to the US cohort. For example, Ghanaians weighed 20.66kg (16.96–24.36kg) less on average than those from the US, while participants from the Seychelles weighed 12.92kg (9.25–16.59kg) less on average.

### Associations between sedentary behaviour and weight change

The GEE models evaluating the association between sedentary behaviour at both timepoints, and weight change are presented in Supplementary Table 5. The interaction term between time and sedentary behaviour was, similarly, not statistically significant (p = 0.125), suggesting that the extent to which weight changed over time was not related to the extent to which sedentary behaviour changed over time (Model 2a). Sedentary behaviour at a particular time was not significantly associated with weight at that same time (p = 0.431) on average (Model 2b). Additionally, on average, weight increased by 2.77kg (2.11–3.44kg increase; p < 0.001) at follow-up, irrespective of sedentary behaviour at any time point.

## Discussion

The current study sought to determine the associations between objectively measured PA and prospective weight change in five diverse African-origin populations. The main finding is that while MVPA at baseline and follow-up was associated with lower body weight, overall MVPA (both at baseline and change from baseline to follow-up) did not impact longitudinal weight changes. Instead, only time, gender, adiposity status and being from the US, were significantly associated with prospective weight change. Similar findings were found when using sedentary behaviour as the main exposure (Supplementary Table 5).

This adds to a growing body of literature exploring the relationship between PA and prospective weight change [15–24, 41, 42]. Some studies, mainly interventions and clinical trials, which similarly used objectively measured PA, have found a positive association between PA and weight change. However, these findings are generally modest, with limited translatability to real-life settings since PA was generally assessed under strict intervention conditions and for relatively short periods of time [22–24]. On the other hand, other longitudinal studies, using subjective self-report to estimate PA, have shown that PA is associated with prospective weight change, such that higher levels of self-reported PA are associated with less weight gained [20, 21, 43]. In contrast, our findings are corroborated in a number of other studies also utilising objectively measured PA [15, 16], including a longitudinal study by Ekeland et al. [44], who similarly did not find an association between change in MVPA over time and body weight over time but did find a reverse association when using MVPA as the outcome measure. While the analyses in this current study adjusted for obesity, future studies should additionally explore the potential reverse impact of body weight (and/or obesity) on MVPA over time, across diverse populations [44]. This would help to clarify lingering questions in the PA-weight change debate. Nevertheless, in addition to MVPA at baseline, this study confirms in a cohort of African-origin populations, that change in MVPA over time is not significantly associated with prospective weight change, either.

The second finding is that overall, irrespective of research site, objectively measured MVPA levels were exceptionally low. In fact, because they were so low, it was necessary to use the 1-min bout data as opposed to the 10-min bout data, because so few people within each site met the 10-min bout threshold. The new WHO 2020 guidelines state that MVPA bouts of any duration now count towards total MVPA volume, irrespective of bout length [8]. This is different from the 2010 guidelines which indicated that MVPA needed to be accumulated in 10-min bouts [6]. This raises questions regarding the impact of these shorter bouts of MVPA on prospective weight change over time. However, since the outcome was weight, it is not possible to draw conclusions about the other measures of cardiorespiratory fitness or cardiovascular health. A study by Ma et al. [45] supports this approach, as they confirm that low levels of MVPA are common, and that shorter bouts can contribute to overall PA levels. Although cardiorespiratory fitness is without a doubt a predictor of overall morbidity and mortality [46, 47], the Ma et al study highlights that while shorter bouts may improve some health markers, longer, more structured MVPA sessions are more beneficial for cardiorespiratory fitness and reducing mortality [45].

Consequently, these results do not suggest that PA is not vital for other health benefits, including cardiovascular health. Even in the current study, weight, was on average lower in those participants who did more MVPA compared to those who did less, or even no MVPA. Non-communicable diseases (NCDs), including Type II diabetes (T2D), heart disease, hypertension, stroke, and breast and colon cancer are increasing globally [48], and research suggests that limited PA and physical inactivity is a contributing factor [19]. Regular PA which has been shown to prevent and improve management of NCDs and cardiovascular diseases such as those mentioned above [5, 6, 11–14], also plays a role in in improving mental health, quality of life, and well-being [5, 6]. Conversely, physical inactivity is linked with the onset of NCDs which, in turn, can result in health problems and all-cause mortality, but it is very importantly also a modifiable primary health risk factor for NCDs in all age groups [6, 49]. Nevertheless, our findings, which contribute to the ongoing debate surrounding PA and prospective weight change, suggest that objectively measured PA is not a good indicator of longitudinal weight change at a population level. The impact of participants’ intention to lose weight and the impact of deliberate MVPA, with the goal of weight loss, remains a topic to be explored in future studies.

The importance of evidence-based public health campaigns is highlighted by our study’s findings, which clearly show the rising levels of obesity across the epidemiologic transition. In low and low-to middle-income countries, there has been a movement of inhabitants to urban centers which has provided access to a mechanized lifestyle and cheaper, often more highly processed energy sources, encouraging the rapid increase in the obesity crisis [15]. The greatest increases in obesity were shown in the countries at the lower HDI end, specifically among the Ghanaian, South African and Jamaican men, where the obesity prevalence doubled over time. The prevalence of obesity in the women is alarmingly high when compared to the men, with at least a third of all women from each site classifying as obese. Similar results were found in the US National Health and Nutrition Examination Survey (NHANES) 2005–2014, which found a higher prevalence of obesity among women compared to men [50]. Having confirmed this pattern in African-origin populations across the epidemiologic transition, global public health efforts are needed to specifically address this risk, across the life course, in women.

Several strengths of the study include the use of longitudinal objectively measured PA, and under-studied population. To minimize measurement errors, the same brand/model of calibrated equipment items were used at all research sites. However, the study also had limitations. While the local communities were representative of their respective local communities, participants may not be representative of the countries in which they live. Because of this, results must be interpreted with caution when considering them across the human development spectrum. Secondly, due to the nature of the study, measurements were completed during different seasons at the different study sites [16], which may have impacted the PA results. Thirdly, sensitivity analysis showed that age was statistically different between groups with and without valid follow-up data, but age was not found to be significantly associated with prospective weight change in the GEE models and therefore we don’t expect that this would have a large impact on the results. Fourthly, the current analysis did not include total or light PA data, which may be useful as per the updated WHO PA recommendations [8]. Fifth the study only analysed daytime PA data between 07:00 and 23:00, assuming this to line up with participants’ wake time. Since it is possible that this period includes some sleep time, we acknowledge that the PA data may include more sedentary behaviour than intended. Lastly, the analysis did not include nutrition data, which is expected to vary widely between sites and is highly relevant when exploring prospective weight change.

## Conclusion

The results of this study indicate that objectively measured MVPA is not associated with prospective weight change over time in 5 African-origin cohorts. Instead, sex, obesity status, and site (a surrogate for stage of the epidemiologic transition) were associated with prospective weight change. While there is much evidence to support the health benefits from engaging in higher levels of MVPA, including lower cardiovascular disease like hypertension, the direct weight loss effects are likely more nuanced. Thus, studies should focus on understanding the drivers of longitudinal weight gain and obesity at the population level, including the obesogenic food environment and social determinants of health.

## Supplementary Material

Supplement 1

## Figures and Tables

**Table 1:** Participant characteristics by site, sex, and time point (n = 851)

	Ghana		South Africa		Jamaica		Seychelles		United States	
Men										
	Baseline	Follow-up	Baseline	Follow-up	Baseline	Follow-up	Baseline	Follow-up	Baseline	Follow-up
Sample size	n = 65		n = 55		n = 55		n = 139		n = 48	
Age (y)	38 (33,42)	46 (41, 50)	34 (30, 38)	42 (38, 46)	37 (31, 40)	45 (39, 48)	37 (33, 41)	45 (41, 48)	39 (35, 43)	46 (42, 51)
Weight (kg)	64 (59, 68)	65 (59, 76)	63 (57, 69)	64 (56, 73)	70 (64, 79)	74 (64, 84)	80 (70, 90)	85 (73, 97)	87 (76, 109)	85 (74, 101)
Height (cm)	167 (161, 174)	167 (162, 173)	171 (167, 174)	171 (167, 175)	176 (172, 179)	176 (173, 180)	174 (169, 178)	174 (170, 178)	176 (172, 180)	176 (171, 179)
BMI (kg/m^2^)	22 (20, 25)	23 (21, 27)	21 (20, 23)	22 (19, 24)	23 (21, 25)	24 (21, 26)	27 (23, 30)	28 (24, 31)	30 (25, 33)	28 (24, 34)
Waist circumference (cm)	78 (74, 83)	85 (78, 94)	79 (73, 84)	81 (74, 89)	77 (72, 87)	82 (76, 94)	89 (82, 98)	95 (88, 103)	97 (85, 112)	98 (89, 110)
Hip circumference (cm)	93 (88, 98)	94 (89, 100)	93 (89, 96)	95 (90, 98)	93 (89, 99)	96 (90, 102)	102 (96, 109)	106 (100, 113)	111 (102, 120)	100 (93, 109)
Fat mass (kg)	11 (8, 14)	13 (10, 20)	14 (10, 17)	18 (14, 22)	13 (9, 20)	16 (10, 24)	20 (15, 26)	25 (20, 33)	28 (18, 41)	30 (23, 39)
Body fat (%)	17 (13, 21)	22 (16, 27)	22 (18, 26)	29 (24, 32)	19 (15, 24)	22 (17, 30)	25 (21, 30)	29 (26, 35)	32 (24, 38)	36 (32, 41)
Overweight	16 (25)	22 (34)	4 (7.3)	8 (15)	14 (25)	15 (27)	57 (41)	50 (36)	12 (25)	18 (38)
Obese	2 (3)	4 (6)	1 (2)	2 (4)	2 (4)	4 (7)	30 (22)	47 (34)	24 (50)	17 (35)
Education (y)	9 (9, 10)	9 (9, 10)	10 (8, 11)	10 (9, 11)	11 (9, 11)	11 (9, 11)	13 (11, 14)	13 (11, 14)	13 (12, 14)	13 (12, 14)
Employed	65 (100)	64 (98)	50 (91)	36 (65)	51 (93)	54 (98)	135 (99)	128 (92)	36 (75)	43 (90)
Manual labour	40 (62)	30 (46)	50 (93)	24 (44)	36 (72)	17 (31)	64 (58)	19 (14)	24 (56)	15 (31)
Women										
Sample size	n = 119		n = 68		n = 51		n = 185		n = 66	
Age (y)	36 (29, 41)	44 (38, 50)	36 (29, 39)	44 (36, 48)	38 (32, 42)	46 (39, 50)	36 (31, 41)	44 (38, 49)	37 (31, 41)	44 (39, 49)
Weight (kg)	63 (56, 71)	72 (62, 80)	79 (66, 96)	83 (67, 106)	74 (61, 84)	82 (65, 91)	69 (58, 79)	76 (66, 88)	97 (77, 111)	97 (83, 113)
Height (cm)	159 (155, 163)	159 (155, 164)	159 (156, 165)	159 (155, 163)	162 (158, 167)	162 (157, 167)	161 (157, 166)	162 (158, 166)	164 (160, 168)	164 (160, 169)
BMI (kg/m^2^)	25 (22, 28)	28 (24, 31)	32 (26, 37)	32 (27, 39)	29 (23, 32)	32 (26, 34)	27 (23, 30)	30 (26, 34)	35 (29, 41)	36 (30, 42)
Waist circumference (cm)	83 (76, 93)	95 (86, 103)	96 (85, 108)	97 (89, 112)	93 (79, 97)	98 (87, 106)	85 (79, 95)	93 (85, 102)	104 (90, 116)	109 (97, 123)
Hip circumference (cm)	101 (93, 109)	105 (98, 114)	112 (104, 122)	115 (99, 127)	106 (96, 112)	107 (99, 115)	102 (97, 111)	108 (100, 116)	121 (107, 131)	123 (107, 132)
Fat mass (kg)	22 (17, 29)	27 (20, 33)	34 (27, 46)	38 (29, 54)	29 (21, 36)	34 (27, 41)	26 (20, 33)	31 (24, 38)	46 (31, 55)	46 (35, 55)
Body fat (%)	35 (31, 39)	38 (35, 41)	44 (39, 49)	48 (44, 52)	41 (36, 43)	43 (38, 46)	38 (34, 42)	41 (37, 45)	46 (42, 49)	48 (42, 52)
Overweight	42 (35)	42 (35)	16 (24)	13 (19)	12 (24)	8 (16)	63 (34)	55 (30)	13 (20)	13 (20)
Obese	20 (17)	42 (35)	37 (54)	41 (60)	22 (43)	31 (61)	50 (27)	86 (46)	46 (70)	49 (74)
Education (y)	9 (6, 9)	9 (5, 9)	10 (9, 11)	10 (9, 11)	11 (10, 11)	11 (10, 11)	13 (11, 14)	13 (12, 14)	14 (13, 16)	14 (12, 16)
Employed	112 (94)	99 (83)	52 (76)	26 (38)	36 (72)	40 (78)	175 (97)	179 (97)	56 (85)	55 (83)
Manual labour	107 (92)	23 (19)	54 (93)	2 (2.9)	29 (66)	2 (3.9)	39 (27)	10 (5.4)	21 (36)	8 (12)

Data are presented as median (interquartile range) or counts (percentage). BMI: body mass index.

Generalised Estimating Equations (GEEs).

**Table 2:** Participants accelerometer-derived PA data, by site, sex, and time point (n = 851)

	Ghana		South Africa		Jamaica		Seychelles		United States	
Men										
	Baseline	Follow-up	Baseline	Follow-up	Baseline	Follow-up	Baseline	Follow-up	Baseline	Follow-up
Individuals with valid PA data	n = 65		n = 55		n = 55		n = 139		n = 48	
Sedentary time (min/d in 1-min bouts)	188 (165, 224)	246 (202, 309)	208 (179, 237)	184 (150, 218)	235 (214, 277)	241 (196, 296)	196 (168, 238)	259 (207, 307)	210 (171, 245)	194 (161, 275)
MVPA (min/d in 1-min bouts)	41 (26, 62)	38 (22, 56)	52 (36, 78)	41 (31, 65)	24 (14, 38)	25 (11, 49)	32 (17, 47)	23 (10, 39)	22 (11, 42)	15 (8, 33)
Sedentary time (min/d in 10-min bouts)	41 (23, 63)	129 (84, 216)	41 (25, 62)	75 (47, 106)	74 (52, 100)	120 (73, 168)	57 (38, 80)	129 (82, 187)	43 (27, 67)	84 (44, 153)
MVPA (min/d in 10-min bouts)	18 (7, 34)	15 (6, 31)	27 (14, 46)	18 (11, 34)	7 (0, 16)	7 (1, 21)	12 (3, 27)	6 (0, 16)	7 (0, 18)	3 (0, 17)
Women										
Individuals with valid data	n = 119		n = 68		n = 51		n = 185		n = 66	
Sedentary time (min/d in 1-min bouts)	194 (169, 218)	270 (217, 334)	216 (197, 249)	185 (144, 218)	211 (174, 242)	202 (174, 234)	185 (152, 219)	251 (201, 304)	211 (179, 240)	190 (156, 225)
MVPA (min/d in 1-min bouts)	23 (14, 36)	16 (8, 30)	20 (11, 29)	14 (8, 25)	17 (8, 33)	13 (8, 19)	22 (13, 32)	16 (8, 25)	7 (4, 16)	8 (2, 15)
Sedentary time (min/d in 10-min bouts)	40 (26, 53)	164 (112, 228)	59 (34, 85)	75 (52, 109)	51 (26, 66)	88 (67, 129)	44 (24, 74)	122 (75, 198)	42 (33, 58)	67 (46, 112)
MVPA (min/d in 10-min bouts)	8 (4, 16)	4 (0, 10)	9 (3, 16)	5 (2,10)	8 (2, 16)	4 (2, 8)	8 (2, 16)	4 (0, 11)	0 (0, 4)	0 (0, 5)

Data are presented as median (interquartile range) or counts (percentage). PA: physical activity; MVPA: moderate-to-vigorous intensity PA.

**Table 3:** GEE linear regression models showing the association between PA and weight over time:

	Model 1a: multivariable with interaction		Model 1b: multivariable without interaction	
	ß estimate (95% CI)	p-value	ß estimate (95% CI)	p-value
MVPA (30min/day)	−0.29 (−0.99; 0.41)	0.41	−0.63 (−1.22; −0.04)	0.04
Sex (female)	−3.66 (−5.55; −1.78)	< 0.001	−3.71 (−5.59; −1.83)	< 0.001
Age (y at baseline)	0.04 (−0.12; 0.21)	0.60	0.04 (−0.12; 0.21)	0.560
Obese	18.17 (16.57; 19.76)	< 0.001	18.20 (16.61; 19.79)	< 0.001
Time (follow-up)	3.11 (2.14; 4.08)	< 0.001	2.61 (2.02; 3.20)	< 0.001
Site (in reference to US)				
Ghana	−20.66 (−24.36; −16.96)	< 0.001 ***	−20.61 (−24.31; −16.91)	< 0.001
South Africa	−15.75 (−20.15; −11.35)	< 0.001 ***	−15.70 (−20.09; −11.30)	< 0.001
Jamaica	−14.77 (−19.30; −10.25)	< 0.001 ***	−14.76 (−19.29; −10.24)	< 0.001
Seychelles	−12.92 (−16.59; −9.25)	< 0.001 ***	−12.89 (−16.56; −9.22)	< 0.001
MVPA and time (follow-up)				
MVPA:time	−0.53 (−1.28; 0.22)	0.16		

MVPA: moderate-to-vigorous intensity PA; CI: confidence interval

Data are presented as median, interquartile ranges, maximum and minimum. MVPA: moderate-to-vigorous intensity PA; GH: Ghana, RSA: South Africa, JA: Jamaica, SEY: Seychelles, USA: United States.

Data are presented as median, interquartile ranges, maximum and minimum. MVPA: moderate-to-vigorous intensity PA; GH: Ghana, RSA: South Africa, JA: Jamaica, SEY: Seychelles, USA: United States.

## Data Availability

Data are available upon reasonable request from the corresponding author.
