# Supplementary material for: Objectively measured moderate-to-vigorous physical activity does not attenuate prospective weight gain among African-origin adults spanning the epidemiological transition
Source: Res Sq. 2024 Dec 16:rs.3.rs-5043485. Preprint. [Version 1] doi: 10.21203/rs.3.rs-5043485/v1 (PMC11702839; doi:10.21203/rs.3.rs-5043485/v1)
Supplement: Supplement 1 [file NIHPPRS5043485v1-supplement-1.pdf]

Supplementary Files

This is a list of supplementary files associated with this preprint. Click to download.

- METSMVPAandprospectiveweight06092024finalsupplementaryinformation.docx
